# Understanding mechanisms of multi-level implementation strategies for autism interventions in a randomized trial across service systems

**DOI:** 10.1186/s13012-025-01466-z

**Published:** 2025-12-15

**Authors:** Aubyn C. Stahmer, Anna S. Lau, Scott Roesch, Elizabeth Rangel, Gregory A. Aarons, Lauren Brookman-Frazee

**Affiliations:** 1 Department of Psychiatry and Behavioral Sciences, MIND Institute, University of California, Davis, 2825 50th St., Sacramento, CA 95819 USA; 2 Psychology Department, University of California Los Angeles, Box 952563, Los Angeles, CA 90095 USA; 3 Department of Psychology, San Diego State University, San Diego, CA 92182-4611 USA; 4 Deparment of Psychiatry, University of California, San Diego, 9500 Gilman Drive, La Jolla, CA 92093-0812 USA

**Keywords:** Implementation strategies, Implementation mechanisms, Evidence-based autism interventions, Mental health, Education

## Abstract

**Background:**

Understanding the effectiveness of implementation strategies to support uptake of evidence-based interventions (EBIs) requires examining activation of mechanisms targeted by implementation strategies. This study uses data from the TEAMS (Translating Evidence-Based Interventions for Autism) hybrid type III implementation-effectiveness trial to examine whether leader-level and provider-level implementation strategies, when paired with provider training in AIM HI (An Individualized Mental Health Intervention for Autism) in mental health programs (Study 1) and CPRT (Classroom Pivotal Response Teaching) in schools (Study 2) successfully activated proposed implementation mechanisms (3 for leader level strategy and 2 for the provider-level strategy). We also examined whether any of the identified mechanisms associated with the leader-level strategy mediated the previously reported effect of the strategy on implementation and child outcomes.

**Methods:**

Organizations were randomized to receive a leader-level strategy (TEAMS Leadership Institute [TLI]), provider strategy, both strategies, or neither strategy (EBI provider training only). Leader participants were recruited from enrolled programs/districts and then supported recruitment of provider/child dyads. Children ranged in age from 3 to 13 years. The combined sample included 65 programs/districts, 95 TLI leaders, and 385 providers/child dyads. Multi-level modeling was used to test hypotheses. The hypothesized mechanisms were implementation leadership, implementation climate, and implementation support strategies for TLI and EBI attitudes and motivation for training for TIPS.

**Results:**

The leader-level strategy engaged the most proximal of the three hypothesized mechanisms (implementation support strategies). The provider-level intervention did not engage any of the hypothesized mechanisms. There was an interaction between the leader-level and provider-level strategies on implementation climate and provider motivation mechanisms favoring groups that received both implementation strategies compared to those that only received the provider-level strategy. No mechanisms significantly mediated the effect of the leader-level strategy on implementation or clinical outcomes.

**Conclusions:**

This study provides support that a brief implementation leadership and climate training, TLI, increases leader use of specific actions to promote autism EBIs across two public service systems, children’s mental health and public education. This does not fully account for strategy effects on fidelity or clinical outcomes. Findings advance the study of implementation mechanisms by examining how leadership training might work and identifying a clear need to focus on leader-level implementation strategies in these systems of care.

**Trial registration:**

ClinicalTrials.gov Identifier: NCT03380078.

Contributions to the literature

- This is the first study to examine the engagement of organizational implementation strategy mechanisms when paired with evidence-based autism interventions.
- We found that a brief leader-level training leads to greater use of implementation support strategies after 6 months, however it did not have measurable effects on implementation leadership or climate broadly.
- The unique design allowed examination of the effects of leader-level and provider-level implementation strategies on their proposed mechanisms.
- The interaction between the provider motivational strategy and the leader-level strategy suggests that leader training may be especially important when providers are not highly motivated for training.

## Background

The rising number of autistic children and the lack of equity in accessing high quality care remains a significant public health challenge. Children’s mental health and the public school system provide the majority of services for autistic children, especially for those in traditionally underserved communities [1]. Multiple evidence-based interventions (EBI) have been developed to support autistic children [2–4]. However, uptake into public systems has been slow and often unsuccessful [5, 6].

Intervention fidelity, or adherence to EBI methods, has been variable when EBIs are scaled in community service systems. Fidelity is important because of its positive relationship with child outcomes [7–9]. To overcome challenges with effective translation of complex EBI into public systems, researchers have been examining implementation strategies to increase their successful uptake [10, 11]. These include implementation strategies that can engage specific mechanisms known to account for variation in EBI outcomes.

Our research teams identified two potential implementation determinants (Leadership support & provider motivation) to target to facilitate the uptake of autism EBIs in schools and mental health clinics (including school-based mental health) based on the literature and data from our Hybrid Type 1 effectiveness trials [12]. These prior independent effectiveness trials tested two collaboratively adapted autism EBI. The first, AIM HI (An Individualized Mental Health Intervention for Autism) for use in publicly funded mental health clinics led to improved behavioral outcomes for autistic children ages 5–13 being seen for mental health concerns by providers with limited autism experience [10]. The second, CPRT (Classroom Pivotal Response Teaching), improved student engagement and behavior for students ages 3–12 in special education classrooms [11]. In both trials, there was variation in provider EBI fidelity and child outcomes were significantly predicted by provider fidelity. Guided by the Exploration, Preparation, Implementation, Sustainment (EPIS) framework [13, 14], mixed methods findings indicated that two inner context implementation determinants appeared to affect EBI implementation in both settings: leadership support and provider motivation [12].

Leadership has been identified as an important implementation mechanism in multiple settings serving children including community mental health [15, 16] and schools [17, 18]. Implementation leadership, a strategic approach to leadership that involves a set of influencing behaviors (e.g., having EBI knowledge, planning training, persisting through challenges etc.) to achieve positive outcomes for implementation, plays a key role in the success of EBI implementation [6, 19, 20]. Leadership, in turn, affects implementation climate (the extent to which an EBI is expected, supported and rewarded) in mental health settings [15] and schools [21–23]. Implementation leadership and climate are often characterized through specific strategies that support EBI implementation, such as talking about the EBI in staff meetings, providing resources for training, or recognizing and rewarding quality EBI use. Williams and colleagues (2022) examined use of three autism EBI in schools. Data supported a pathway from increased implementation leadership to higher implementation climate and subsequently higher fidelity to one EBI [24]. Implementation climate has also been linked to increased EBI fidelity, EBI sustainment, and improved child outcomes in both setting types [25–29].

Leadership interventions have been used to improve EBI implementation in mental health and school settings in large service systems. One comprehensive program, Leadership and Organizational Change for Implementation (LOCI) [16] focuses on a range of leadership skills for first-level leaders and has been tested in public mental health and substance use disorder treatment systems and has been deployed in organizational, countrywide, and statewide implementation efforts [30–32]. LOCI has been perceived by leaders as having high utility in improving statewide EBI implementation [33], results in improved provider perceptions of implementation and implementation climate [30], and improved EBI fidelity compared to controls in youth mental health clinics [31]. Finally, recent data supports the implementation logic model that engagement of implementation climate in LOCI accounts for variation in provider fidelity to measurement-based care in youth mental health services [34].

Provider motivation to implement an EBI, often measured by attitudes toward EBI adoption or behavioral intentions to use EBI, has been posited as important for implementation in multiple systems of care [35, 36]. Provider motivation and/or attitudes have recently been linked to successful use of EBI in schools [27] and mental health clinics [37]. In a study of an implementation strategy which included motivational interviewing as one component of provider training, clinicians in the experimental group stayed in consultation up to four times longer than those in the control group. [38]. In a similar study with an adapted model specifically for teachers, fidelity improved for all participants with a marginal trend in favor of the implementation strategy [39]. However, few studies have examined implementation strategies to change provider motivation and attitudes toward EBI and it has yet to be demonstrated that engaging provider motivation mechanisms can improve provider implementation outcomes.

Based on our data and the literature we tested the effectiveness of two implementation strategies, one targeting implementation leadership and climate and one targeting provider motivation (or their combination) on improving provider EBI fidelity and child clinical outcomes when accompanying CPRT in schools or AIM HI in publicly funded mental health clinics [40]. The leader-level strategy, Teams Leadership Institute (TLI; described below) led to significantly improved provider EBI fidelity and child behavioral outcomes [40]. This is consistent with other literature using similar models reporting higher EBI fidelity in groups receiving leadership training compared to control conditions [31]. However, we did not find any significant differences between groups based on participation in the provider-level motivational strategy, TEAMS Individual Provider Strategy (TIPS) for training.

Most studies focus on how implementation strategies affect implementation outcomes (e.g., fidelity, acceptability, reach) or patient/student outcomes (e.g., mental health, communication, treatment compliance). To better understand how and why these implementation strategies work, or do not work to address implementation outcomes, there has been a strong call to examine the effects of these strategies on implementation mechanisms [41, 42]. Examining mechanism engagement is a necessary step in advancing our understanding of implementation strategy effectiveness [41–43]. Implementation mechanisms have been defined by Lewis et al., 2018 [34] as the processes or events through which implementation strategies operate to support positive outcomes. Recently there has been a strong push to study implementation mechanisms to better understand the impact of implementation strategies in community settings, and especially to support adaptation of these strategies based on contextual factors [42].

Mechanisms have been examined in two prior studies of leadership and provider attitude interventions. In a trial in youth mental health, organizations who received LOCI improved their implementation leadership and implementation climate from baseline to follow up with large effects, but LOCI had smaller effects on transformational leadership [44]. A motivational intervention for teachers had the intended effects on the identified mechanisms by either improving some aspects of provider attitudes or preventing deterioration [44]. The largest effects related to task self-efficacy and intentions to implement [44]. To our knowledge, no study has examined implementation mechanisms to support uptake of autism EBI.

**Fig. 1:**
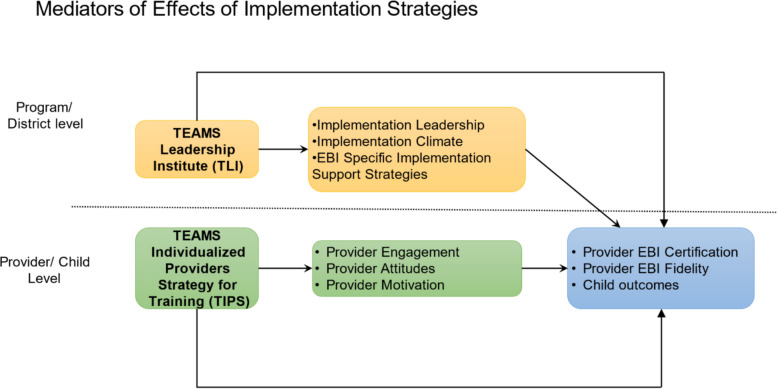
Mechanisms

The current study has two primary aims. Aim 1: to examine the impact of the implementation strategies, TLI and TIPS, on targeted leader- and provider-level mechanisms of change (See Fig. 1). We hypothesize that (1a) TLI will increase implementation leadership and climate and the use of implementation leadership strategies and (1b) TIPS will lead to greater change in provider attitudes and engagement in EBI training. Aim 2: to examine if mechanism engagement can explain the observed effects of the TLI strategy on provider EBI fidelity and child outcomes.

## Methods

The TEAMS project combined two linked trials to examine implementation strategies across EBI and setting. Eligibility differed by study. The *AIM HI Study* (Study 1) included California mental health programs that provided publicly funded psychotherapy services to autistic children ages 5–13. The *CPRT Study* (Study 2) included school districts that provided public education services to students in preschool through fifth grade. After programs/districts enrolled, they were randomized to Standard Training, TLI, TIPS or combined TLI and TIPS enhanced training. Study data collection for both studies were coordinated and pooled for analyses. Randomization occurred at the program/district level prior to any participant enrollment to prevent contamination at the provider level and was stratified by California County (San Diego, Sacramento, Los Angeles, Other) using randomly permuted blocks according to a computer-generated assignment sequence prepared in advance by the study statistician. The University of California, San Diego Institutional Review Board approved this study, and informed written consent was obtained from all participants or their guardians.

## Participants

All participants were recruited from eligible, enrolled programs and school districts. The full study consort diagram is available in Brookman-Frazee et al., 2025 [40]. The combined sample included 65 programs/districts, 95 leaders, 387 providers and 385 children. See Table 1 for a summary of participant characteristics.

**Table 1:** Leader, provider, and family characteristics

Study Intervention	Total	AIM HI	CPRT
	n (%)		
Leader Characteristics (TLI Participants)	95	50	45
Age, y, *mean (SD)*	44.8 (10.7)	44.5 (11.8)	45.3 (9.4)
Sex (female)			
Female	62 (74.7)	31 (70.5)	31 (79.5)
Male	21 (25.3)	13 (29.5)	8 (20.5)
Ethnicity			
Hispanic/Latino	22 (23.2)	16 (32.0)	6 (13.3)
Non-Hispanic/Latino	61 (64.2)	28 (56.0)	33 (86.7)
Not reported	12 (12.6)	6 (12.0)	6 (13.3)
Race			
Asian	2 (2.1)	1 (2.0)	1 (2.2)
Black or African American	2 (2.1)	2 (4.0)	0
Hawaiian and Pacific Islander	0	0	0
More than one race	8 (8.4)	6 (12.0)	2 (4.4)
Not reported	12 (12.6)	6 (12.0)	6 (13.3)
Other	7 (7.4)	6 (12.0)	1 (2.2)
White	64 (67.4)	29 (58.0)	35 (77.8)
Education			
Bachelor’s	8 (9.5)	3 (6.7)	5 (12.8)
Master’s	67 (79.8)	36 (80.0)	31 (79.5)
Doctoral	9 (10.7)	6 (13.3)	3 (7.7)
Provider (Therapist/Teacher) Characteristics	387	190	197
Age, y, *mean (SD)*	36.3 (9.4)	33.6 (8.08)	39.1 (9.8)
Sex			
Female	351 (91.2)	162 (85.3)	189 (96.9)
Male	34 (8.8)	28 (14.7)	6 (3.1)
Ethnicity			
Hispanic/Latino	110 (28.4)	81 (42.6)	29 (14.7)
Non-Hispanic/Latino	275 (71.1)	109 (57.4)	166 (84.3)
Not reported	2 (0.5)	0 (0.0)	2 (1.0)
Race			
American Indian or Alaskan Native	3 (0.8)	3 (1.6)	0 (0.0)
Asian	24 (6.2)	9 (4.7)	15 (7.6)
Black or African American	9 (2.3)	8 (4.2)	1 (0.5)
Hawaiian and Pacific Islander	2 (0.5)	0	2 (1.0)
More than one race	26 (6.7)	14 (7.4)	12 (6.1)
Not reported	3 (0.8)	0 (0.0)	3 (1.5)
Other	46 (11.9)	37 (19.5)	9 (4.6)
White	274 (70.8)	119 (62.6)	155 (78.7)
Education			
Bachelor’s	103 (26.6)	15 (7.9)	88 (44.7)
Master’s	255 (65.9)	157 (82.6)	98 (49.7)
Doctoral	16 (4.1)	16 (8.4)	0
Primary Professional Discipline			
Special Education	150 (38.8)	0.0	151 (76.6)
Marital and family therapy	102 (26.4)	102 (53.7)	0
Social work	47 (12.1)	47 (24.7)	0
Clinical psychology	22 (5.7)	22 (11.6)	0
Speech and language pathology	15 (3.9)	0	15 (7.6)
Education	12 (3.1)	0	12 (6.1)
School psychology	9 (2.3)	7 (3.7)	2 (1.0)
Psychiatry	5 (1.3)	5 (2.6)	0.0
Behavior analysis	4 (1.0)	0	4 (2.0)
Mental health counselor	4 (1.0)	4 (2.1)	0
Occupational therapy	4 (1.0)	0	4 (2.0)
Other	2 (0.5)	1 (0.5)	1 (0.5)
Years practiced, *mean (SD)*	6.6 (6.1)	5 (4.7)	8.8 (7.0)
Autism Specialization (yes)	162 (41.9)	26 (13.7)	137 (69.5)
COVID Training Status			
Pre-COVID	229 (59.2)	120 (63.2)	109 (55.3)
COVID disruption	100 (25.8)	38 (20)	62 (31.5)
Post-COVID	58 (15.0)	32 (16.8)	26 (13.2)
Child Characteristics	385	188	197
Age, y, *mean (SD)*	8 (2.9)	9.7 (2.3)	6.3 (2.4)
Sex			
Female	74 (19.9)	42 (22.6)	32 (17.3)
Male	297 (80.1)	144 (77.4)	153 (82.7)
Ethnicity			
Hispanic/Latino	173 (44.7)	107 (56.3)	66 (33.5)
Non-Hispanic/Latino	195 (50.4)	76 (40.0)	119 (60.4)
Not reported	19 (4.9)	7 (3.7)	12 (6.1)
Race			
American Indian or Alaskan Native	4 (1.0)	1 (0.5)	3 (1.5)
Asian	34 (8.8)	0 (0.0)	34 (17.3)
Black or African American	17 (4.4)	10 (5.3)	7 (3.6)
Hawaiian and Pacific Islander	5 (1.3)	0 (0.0)	5 (2.5)
More than one race	52 (13.4)	29 (15.3)	23 (11.7)
Not reported	39 (10.1)	19 (10.0)	20 (10.2)
Other	33 (8.5)	20 (10.5)	13 (6.6)
White	203 (52.5)	111 (58.4)	92 (46.7)
Caregiver Preferred language (Spanish)	39 (10.1)	30 (15.8)	9 (4.6)
Caregiver Highest level of education			
High school/GED	145 (37.5)	85 (44.7)	60 (30.5)
AA degree	54 (14)	20 (10.5)	34 (17.3)
Bachelor’s	80 (20.7)	27 (14.2)	53 (26.9)
Master’s	28 (7.2)	14 (7.4)	14 (7.1)
Doctoral Degree	5 (1.3)	0 (0)	5 (2.5)
Other	52 (13.4)	36 (18.9)	21 (9.6)

### Leader TLI participants

Leaders from enrolled programs in both studies were eligible to participate when they provided administrative oversight to an enrolled mental health program or school district and their program/district was randomized to TLI. Programs identified leaders based on administrative role, which consisted of more typical leadership roles (e.g., program director, associate directors of special education) or had a supervisory role in the program which often consisted of first level leaders (lead psychologist, program specialists). The leader sample (*n* = 95) was primarily female (74.7%). 67.4% identified as White, 2.1% as Black, 2.1% as Asian, 8.4% as multiracial, and 7.4% as other racial categories. 23.2% of leaders identified as Hispanic or Latino/Latinx. The leader sample was 44.8 years of age on average (SD = 10.70). The majority of leaders had a master’s degree (79.8%). The most common educational disciplines that leaders represented were Marriage and Family Therapy (29.5%) and Special Education (20.4%). The most common leader role title was “Director” (25.9%). Frequent role titles (occurring more than 3 times) included director, supervisor, specialist, coordinator, lead, psychologist, and manager. Lower frequencies were present in roles such as assistant superintendent, therapist, behaviorist, chief, assistant principal, and principal.

### Provider participants

*AIM HI study*. Therapists were eligible when they were employed as staff or a supervised trainee in an enrolled program; anticipated employment/training for at least the next 7 months; had an eligible child on their current caseload; and did not participate in the prior AIM HI trial. *CPRT Study:* Teachers were eligible when employed in an enrolled district; anticipated employment for the academic year; had an eligible child in their classroom; and did not participate in the prior CPRT trial.

### Family participants

Families enrolled as a dyad with a participating provider. *AIM HI Study:* Eligible families had a child aged 5 to 13 years old; child had an existing autism diagnosis on record; child and parent spoke English or Spanish as their primary language. *CPRT Study:* Eligible families had a child aged 3 to 10 years who had a primary educational classification of autism.

Both studies simultaneously began prior to, and continued through, the onset of the COVID-19 pandemic. Providers included those trained in the EBIs before COVID (“Pre-COVID”, i.e. EBI training completed before March 13, 2020 with no changes to training or intervention delivery; *n* = 229), those who were active in training at the time of initial COVID impact (“COVID Disruption” i.e. training and intervention delivery underway on March 13, 2020 requiring changes to training or intervention delivery mid cohort; *n* = 100), and those who began training after COVID onset (“Post-COVID Onset”, i.e. training started August 2020, all training and some intervention conducted remotely for the entire training period; *n* = 58). Chi-squared analyses indicated no differences in age, gender, race or ethnicity based on cohort.

## Procedures

### Clinical Interventions (EBIs) and provider training procedures

Below we provide a brief summary of the clinical interventions and provider training. Additional information can be found in: [10, 11, 40, 45].

AIM HI is a package of evidence-based, parent-mediated and child-focused strategies designed to address interfering behaviors in children with autism ages 5 to 13 years through the development of child and caregiver skills. AIM HI takes approximately six months to deliver and involves a series of protocol steps and session adaptations aimed to teach parents of autistic children skills to manage interfering behaviors and teach children positive alternative skills.

CPRT is a naturalistic behavioral intervention designed to increase learning through improving motivation and engagement. It includes components such as providing clear instructions, sharing control with the child, interspersing easy and difficult tasks, and reinforcing attempts. CPRT includes tools to implement these strategies in the context of classroom activities and to address educational goals.

Provider EBI training was conducted by master’s or doctoral level clinicians employed by the research study with extensive training and experience with the EBIs. Standard training for each EBI included initial didactic workshops, followed by ongoing coaching/consultation for approximately 6 months with case-specific feedback, and performance monitoring via video and material review [46].

## Implementation strategy procedures

### TEAMS Leadership Institute (TLI)

See Table 2 for a description of TLI components [15, 40]. TLI is a data-informed strategy to enhance the effectiveness of provider training in autism EBIs by coaching leaders in the strategic selection of implementation leadership and climate building strategies to meet an organization’s needs. TLI included a 360 assessment of implementation leadership and climate, a pre- implementation workshop with leaders, a data-informed development of an implementation climate and leadership plan, follow-up, bi-weekly coaching calls, a booster session and graduation. Leaders in school districts and publicly funded mental health clinics chose to participate in TLI as leadership teams. On average each team consisted of 3 leaders (M = 3.39, Range: 1–6; SD = 2.20).

**Table 2:** Implementation strategy components

TEAMS Leadership Institute (TLI, Leader-Level Strategy)			
Components	Content	Format/Intensity	Additional Full LOCI Content
360 Assessment	Implementation Climate & Leadership	Leader and provider surveys	Assesses additional domains (e.g., transformational leadership)
Initial training	Implementation Climate & Leadership; Autism EBIs; Use of assessment reports to develop program/district leadership plans	Length: 3 h Participants: teams of leaders from each program/district	Length: 16 h Participants: one first level leader Additional content not in TLI: transformational leadership and personalized leadership development plans
Coaching calls	Track goals and refine leadership plans	Length: 15–30 min Duration: 6 months Frequency: 2 × per month with option to adjust frequency after 4 months Participants = individual leader or teams of leaders from each program/district	Length: 15–30 min Duration: 12 months Frequency: weekly with monthly group call with first level leaders from other programs Participants = one first level leader
Booster session	Review progress and refine goals with a focus on sustainment planning	Length: 2 h Timing: Month 4	Length: 6 h Timing: Months 4 and 8
Graduation	Celebrate and recognize provider completion of EBI training and leader completion of TLI	Timing: Month 6	Timing: Month 12
TEAMS Individualized Provider Strategy (TIPS, Provider-Level Strategy) Version of Evidence-Based Intervention (EBI) Training			
Components		Content Description	
Pre-Workshop Phone Call		TIPS EBI Coaches/Trainers had phone discussion with providers designed to promote change talk around learning and using the EBI	
Initial EBI Training Workshop Enhancements		Embedded discussion points designed to elicit and reinforce change talk, emphasize autonomy, and affirm strengths and change efforts	
EBI Coaching/Consultation Enhancements		Embedded reflective methods to facilitate provider independence, relate training to broader professional goals, reflect on provider confidence in and commitment to using the strategies, and collaboratively plan for next steps	
Motivational messages between coaching sessions/consultations		Reminder emails used to support EBI delivery between coaching sessions/consultations adapted to include motivational messages to encourage ongoing participation	
Proactively identified rescue strategies		Coaches/trainers identified rescue strategies (e.g., additional email or call) to support change efforts when needed	

The targeted mechanisms for TLI are Implementation leadership, implementation climate. The targeted mechanism for TIPS is provider motivation for training. *LOCI* Leadership and Organizational Change for Implementation

During an initial 3-h workshop leaders received training in implementation leadership and climate, a summary of the EBI and the rational for EBI use in publicly-funded programs, and reviewed their organizations 360 data. Leaders worked with their TLI coach to identify implementation leadership and climate goals and objectives and to develop their plan. Leaders engaged in regular coaching calls of 15–30 min approximately bi-weekly to plan and problem solve implementation issues. The was an average of 8.7 coaching calls per program/district (SD = 2.69, range = 3–15). After approximately 4 months, leaders participated in a 2-h booster workshop to examine progress toward goals, effectiveness of the strategies they identified, and to develop an EBI sustainment plan. Finally, leaders and providers participated in a joint graduation at EBI training completion.

TLI training was conducted by masters or doctoral level clinicians (primarily post doctoral fellows) with training and experience with the two autism EBIs. Most had some experience working in public service systems and received training in understanding the structure of each system as part of learning TLI. Model adherence was assessed using a content checklist. None of the Non-TLI programs/districts received the TLI workshop or coaching calls.

### TIPS

TIPS integrated motivational interviewing principles and strategies into the EBI provider training. EBI coaches in the TIPS condition received training in the strategies from an expert in motivational interviewing. The goal was to address attitudinal barriers and improve engagement of providers by eliciting and reinforcing change talk [47]. TIPS components consisted of pretraining calls, proactive planning, ongoing reflection, and motivational notes. TIPS coaches utilized reflective methods to address concerns and barriers about implementation and collaborative development of next steps.

AIM HI and CPRT trainers in the TIPS condition received MI training, ongoing coaching, and fidelity monitoring with an expert consultant who supported the development of the TIPS provider training process. See Table 2 for a description of how TIPS training protocols incorporated motivational interviewing. TIPS fidelity was assessed using the 12-item MI Coach Rating Scale (MI-CRS) [48] for all trainers by motivational interviewing experts naïve to condition. TIPS trainers averaged 70% fidelity to TIPS strategies (range 50–100%) whereas Standard (Non-TIPS) Trainers averaged 2% fidelity to TIPS strategies (range 0–17%).

## Measures

### Aim 1 measures

Aim 1 examines the impact of the implementation strategies, TLI and TIPS, on leader and provider level mechanisms of change. Provider-reported scores on the measures below were used to assess these mechanisms.

### TLI potential mechanisms

#### Implementation Leadership Scale (ILS)

The Implementation Leadership Scale (ILS) [49] is a 12-item scale that captures provider perceptions of leader behaviors thought to promote organizational culture and climate for EBP implementation. The ILS has four subscales representing Proactive, Knowledgeable, Supportive, and Perseverant Leadership. The ILS items were scored on a scale of 0 (Not at All) to 4 (To a Very Great Extent) and summed for a total ILS score and calculated for the four subscales. Total scores gathered at baseline and 6 months were used in the analyses. The reliability within our sample at baseline and at 6 months was α = 0.9.

#### Implementation Climate Scale (ICS)

The Implementation Climate Scale (ICS) [25] is an 18-item measure that aims to capture provider perceptions of the presence of a strategic climate for EBI implementation. It captures six dimensions of organizational context that indicate the extent that organizations prioritize and value the successful implementation of EBPs including openness, recognition, selection, focus, educational support, and rewards. The internal consistency reliability for total scores is strong (a = 0.91; [25]. Total scores gathered at baseline and 6 months were used in the analyses. The reliability within our sample at baseline was α = 0.93 and at 6 months was α = 0.94.

#### Implementation Support Strategies (ISS)

The 20-item Implementation Support Strategies (ISS) measures specific, discrete actions program leaders take to support EBI implementation. A preliminary psychometric analysis of this measure was conducted using data from 150 leaders (leader in TLI and non-TLI programs) from 65 programs. A series of exploratory factor analyses using principal axis factoring (PAF) and direct oblimin rotation were used to explore the factor structure of the ISS. The PAF indicated that a 1-factor solution best fit the data with 64.98% of the variance explained and all 20 items with adequate factor loadings (0.71 to 0.88).

The ISS was completed after 6 months of implementation training to assess provider perceptions of implementation strategies utilized by the organization’s leaders to support the implementation of the two autism EBPs. Respondents rated each item on a scale from 0 (Not at all) to 5 (Very great extent). Total scores were used in the analyses. Internal consistency for our sample was α = 0.97.

### TIPS potential mechanisms

#### Provider engagement

Provider engagement in training was measured through training and consultation attendance (trainer rated) and adherence to training requirements. Attendance was calculated by totaling the number of workshop days, consultation and coaching sessions attended. Training requirements included completion of EBI-specific forms submitted to EBI trainers. Percentage scores were examined for attendance and EBI forms.

#### Evidence-Based Practice Attitude Scale (EBPAS)

Provider attitudes were gauged using the Evidence-Based Practice Attitude Scale (EBPAS). [50] The EBPAS has four subscales: 1) ‘requirements’ that captures the chance of adopting an EBP if it is required, 2) ‘appeal’ that gauges the intuitive appeal of an EBP, 3) ‘openness’ captures if providers are open to new EBPs, and 4) ‘divergence’ captures if the EBP is perceived to differ from usual care. Items are rated from 0 (Not at All) to 4 (To a Very Great Extent). The EBPAS total score was computed by summing all items (after reverse scoring the Divergence subscale). The EBPAS has established reliability and validity [51]. and internal consistency for the total score within our sample was α = 0.79.

#### Provider Motivation Inventory (PMI)

The Provider Motivation Inventory (PMI) was adapted from the Parent Motivation Inventory [52]. There are separate versions for baseline (pre training) and 6 months (post training/coaching). The Baseline PMI (PMI – desire to learn) subscale includes 7 items aimed at assessing providers’ motivation to engage in the building the skills required for an EBP (Martinez et al., under review). Respondents rate each item on a scale of 1 (strongly disagree) to 5 (strongly agree). Items are summed to calculate a subscale score. At the 6 month follow up period, we utilized the PMI –motivation to continue subscale and the PMI– ability and impact subscale as these were most relevant post training (RMSEA) = 0.08, Comparative Fit Index (CFI = 0.97); Standardized Root Mean Square Residual (SRMR) = 0.03). Items in the 6 month follow up version of the PMI – motivation to continue use subscale (7 items total) included “I am eager to continue to use the training resources.” The PMI – ability and impact subscale included 3 items (e.g., “I believe that I am capable of using the strategies learned in this intervention”). The reliability within our sample at baseline for PMI – desire to learn was α = 0.80 and at 6 months PMI – motivation to continue use was α = 0.92 and PMI – ability and impact was α = 0.85. Baseline PMI – desire to learn was used as a covariate in analyses of the 6-month PMI data.

### Aim 2 measures

Aim 2 examines whether mechanism engagement can explain the effects of the implementation strategies on provider EBI fidelity and/or child outcomes.

#### Provider fidelity

Provider EBI fidelity was based on ratings by trained observers naive to implementation condition and time frame. Fidelity ratings captured provider use of the core strategies included in the AIM HI and CPRT protocols. After the initial EBI workshops, participating providers submitted recordings during the 6-month training/consultation period*.* Providers submitted a total of 2364 video recordings. Up to three sessions from each provider (1 per 2-month period) were randomly selected for coding for a total of 556 coded sessions. Ninety-three percent of providers had a video from months 1–2, 79% in months 3–4, and 57% in months 5–6. Average EBI Fidelity scores were standardized within each study (z-scores) for use in combined analysis.

#### Study-Specific Child Outcomes (Effectiveness Outcome)

Caregivers in the AIM HI study completed the Eyberg Child Behavior Inventory (ECBI), [53] a caregiver-report measure assessing common behaviors in children aged 2–16 years, and the extent to which caregivers perceive the behaviors as concerning. The ECBI includes 36 items rated on a dichotomous Problem scale and a 7-point Likert Intensity scale. The Intensity scale was used in the current study. The ECBI demonstrates strong psychometric properties, including test–retest reliability (*r* = 0.80) and convergent and divergent validity [53, 54].

In the CPRT study, parents completed the Pervasive Developmental Disorder Behavior Inventory (PDDBI) [55] rating scale which measures maladaptive and adaptive behaviors in children 2–12 years. The measure has good construct, developmental, and criterion-related validity [55]. The Autism Composite T-scores were used in the current study. The PDDBI had good internal consistency in the current trial (α coefficients = 0.91).

Child measures were completed at baseline and 6 months post baseline.

## Analytic plan

Multilevel models using full information maximum likelihood estimation were used to examine the target predictive models. Analyses were performed using the MPlus statistical software. Missing data and deviations from non-normality were accounted for using Maximum Likelihood – Robust approach implemented in MPlus. Training timing relative to COVID-19 pandemic onset (Pre-COVID, COVID Disruption, Post-COVID onset) and study (AIM HI versus CPRT) were examined as potential covariates in preliminary bivariate analyses with each target mechanism variable. Baseline PMI – desire to learn subscale was used as a covariate in models for PMI – motivation to continue use subscale and PMI – ability and impact subscale measured at 6-months. Identified covariates were included in the final predictive models for both Aims.

For Aim 1, analyses proceeded using a 3-level nested data structure [Level 1 = time Level 2 = provider; Level 3 = program/district) for ICS, ILS, EBPAS; or a 2-level nested data structure for all other variables, given that the focus was on the 6-month time-point only for these variables. For 3-level models, all higher-order interaction terms (3-way interaction representing Time X TLI X TIPS and all 2-way interactions) were tested; for 2-level models a 2-way TLI X TIPS interaction effect was tested. If an interaction term was statistically significant, follow-up analyses followed the procedures outlined by Preacher et al., 2006 [56]. All analyses followed the intention-to-treat principle. For Aim 2, multilevel path analysis was used to test for mediated effects. Mediation models were only tested when statistically significant intervention effects on target mechanism variables in Aim 1 analyses. MacKinnon’s asymmetric confidence interval (CI) was used as a formal test for mediation; CIs that do not contain the value 0 are considered statistically significant mediated effects.

## Power analysis

The power analysis programs RMASS2 and PASS were used to estimate the sample size necessary to find statistically significant (alpha = 0.05) implementation condition effects across time. Power analyses for Aim 1 assumed an effect size of d = 0.43 for a target mechanism variable and overall attrition rate of 14%. Moreover, clustering at the program level was accounted for with the design effect (DE = 1.28). Given these estimates, targeted sample size of 400 provider/child dyads in 50 programs/districts achieves 80% power to find a statistically significant effect using an alpha level of 0.05. Lastly, a series of power analyses were conducted to explore effect size estimates for the potential mediation relationships specified in Aim 2. Using the assumptions/estimates outlined above, the Monte Carlo approach outlined by Schoemann and colleagues was used. Within the context of each analysis, 1000 replications with 20,000 Monte Carlo draws per replication was implemented. Power was calculated using a variety of effect sizes to reflect the magnitude of antecedent to mediator path (path a) and the mediator to outcome path (path b). Given the specifications above, 80% power will be achieved for mediation for effect sizes estimates of d = 0.63 for both compound paths that comprise the mediated effect.

## Results

### Aim 1. To examine the impact of the implementation strategies, TLI and TIPS, on targeted leader and provider level mechanisms of change

#### Implementation Leadership Scale (ILS) at baseline and 6 months

There were no significant differences between the TLI and No TLI groups (B = −0.036, *p* = 0.462).

#### Implementation Climate Scale (ICS) at baseline and 6 months

There were no significant main effects of either implementation strategy on the ICS (TLI: B = −0.01, *p* = 0.93, 95% CI = −0.29—0.26; TIPS: (B = −0.08, *p* = 0.57, 95% CI = −0.37—0.20). There was a significant TLI x TIPS x Time interaction for the ICS (B = 0.22, *p* = 0.03, 95% CI = 0.020—0.42, d = 0.46). Follow-up tests found a significant increase in ICS scores from baseline to 6 months for those that received both TIPS and TLI (B = 0.19, *p* < 0.01; *M*s = 2.26 vs. 2.45) relative to those that received TIPS without TLI (B = −0.03, *p* = 0.70; *M*s = 2.67 vs. 2.64).

#### Implementation Support Strategies (ISS) at 6 months

There was a significant main effect of TLI (B = 0.81, *p* < 0.001, 95% CI = 0.51–1.04, d = 1.05), with higher ISS scores at 6 months for programs that received TLI (M = 1.98) than those that did not receive TLI (M = 1.17). We found no significant main effect for TIPS (B = 0.16, *p* = 0.26, 95% CI = −0.120—0.14) and no TIPS X TLI interaction.

### Provider engagement

#### Evidence-Based Practice Attitudes Scale (EPBAS) at baseline and 6 months

There was no significant main effect of TLI (B = 0.03, *p* = 0.72, 95% CI = −0.15—0.28) or TIPS (B = 0.07, *p* = 0.55, 95% CI = −0.15—0.21) and no TIPS x Time interaction for EBPAS scores (B = 0.062, *p* = 0.160; 95% CI = −0.04—0.15) indicating no significant differences between TIPS and No TIPS groups.

### Provider Motivation Inventory (PMI) at 6 months

There was no significant main effect of either implementation strategy on the PMI (TLI: B = −0.07, *p* = 0.23, 95% CI = −0.17—0.04; TIPS: B = 0.06, *p* = 0.28, 95% CI = −0.05—0.17). For PMI-ability and impact, there was a statistically significant 2-way TLI x TIPS interaction (B = 0.24, *p* = 0.020, 95% CI = 0.037—0.44, d = 0.74). Follow-up tests found significantly higher PMI-ability and impact scores at 6 months for those that received both TIPS and TLI (B = 0.14, *p* = 0.048, *M*- 4.01) relative to those that received TIPS but not TLI (B = −0.111, *p* = 0.180, *M* = 3.77). There were no significant associations between PMI – motivation to continue use and either implementation strategy.

### Aim 2: to examine if mechanism engagement can explain the observed effects of the TLI strategy on provider EBI fidelity and child outcomes

No hypothesized mechanisms mediated the effects of TLI on child outcomes or EBI fidelity.

## Discussion

This is one of very few studies examining significant associations between implementation strategies and their mechanisms in public service systems, and the first to do so in relationship to autism EBIs. These data increase the evidentiary basis for specific implementation strategies, which is currently rather sparse. The leader-level implementation strategy, TLI, engaged the most proximal of our three hypothesized mechanisms on its own; programs and districts that received TLI showed greater provider reported leader use of implementation support strategies than those that did not receive TLI. TLI seemed to have a protective effect on the more global measure of implementation climate when paired with TIPS. The provider-level strategy, TIPS, did not engage any of the hypothesized mechanisms in the way they were measured in this study. Interestingly, TIPS and TLI together did show higher provider motivation to sustain the EBI and their perceived ability to use the EBI to impact child outcomes than TIPS without TLI. TLI did not mediate either EBI fidelity or child outcomes.

These findings align with recent studies using the full LOCI strategy in children’s mental health [31] and also provide novel information about how adaptation of an implementation strategy may affect how or if a mechanism is activated [42]. The briefer TLI may be more feasible to implement than the full LOCI model in highly impacted public service systems and still has impact on one targeted mechanisms. However, while we saw activation of a proximal measure of specific implementation strategy use, we did not see activation of implementation leadership or climate as reported by Williams and colleagues [31]. The abbreviated timeframe and fewer observations may explain the different findings and changes in those constructs may take additional time. TLI, like LOCI, is multi component and the current analysis did not allow for determination of the impact of specific components (e.g. training and development of individual leadership goals versus training and development of implementation climate goals for the program). Additionally, it is possible that the lack of differences between conditions on changes in implementation climate and leadership related to the types of leaders included in TLI relative to LOCI. Specifically, we engaged multiple leaders across levels whereas LOCI has traditionally targeted individual mid-level leaders. Children’s mental health and school district leaders requested a team-based participation method which may have increased the focus on implementation support strategies and climate rather than individual implementation leadership goals. Implementation climate goals tend to be more related to the program while leadership goals tend to be more personalized to the individual leader. Understanding the role of the team in leadership training, and how to support teams in being more effective at implementing TLI will be an important next step.

Alternatively, leaders may need additional training in multiple forms of leadership (e.g., transformational) and specific coaching related to their leadership goals which were not included in TLI. Transformational leadership has been linked with attitudes toward EBI in mental health settings [35]. Interestingly, in the Williams study implementation leadership, but not transformational leadership, had a significant indirect effect on implementation climate indicating the importance of implementation leadership. We could not test this relationship as changes in implementation climate and leadership were measured at the same time points. Additionally, time may have been a factor, as measures were conducted after 6months of participation which may not have allowed enough time to support changes in more distal measures.

The TIPS provider level intervention did not itself engage any of the proposed provider motivation mechanisms, which may explain why the condition did not have a significant main effect on implementation or child outcomes [40]. This may be due to the limited dosage of motivational interviewing added to already lengthy provider training. Additionally, the standard, Non-TIPS training already included attention to adult learning strategies to support provider engagement. While the standard trainers had very low fidelity scores with respect to using motivational interviewing strategies, the professionals who signed up to participate in a research study may have already been quite motivated to learn new skills to support autistic children in their care perhaps restricting our range. Testing the effect of specific TIPS components (e.g., motivational emails; rescue strategies) in providers with lower motivation to learn an EBI may be more informative for building an effective motivational package.

Results suggest that TLI may have provided some protective support for providers in the TIPS conditions. Providers in programs and districts in the TIPS only condition reported less improvement ICS scores from baseline to 6 months relative to providers in sites assigned to the TIPS + TLI condition. The reason for this is unclear, but it is possible that something about the TIPS strategy led providers to identify areas of support they needed, but were not receiving, from their leaders and the addition of TLI led to leadership changes that seemed more supportive. Another potential factor may be that both strategies were employed simultaneously. Addressing leader levels issues first and then addressing provider level motivation (or vice versa) may affect outcomes and could be studied in future trials. Although providers reported that their leaders used implementation support strategies in both conditions, those in the TLI condition reported their leaders used significantly more. With TLI, programs provided more strategies aimed at supporting EBI use and sustainment, and providers perceived higher implementation climate. Implementation climate then, may serve to support providers in challenging environments.

Although we found support for the effects of TLI on ISS, we did not find support that any of the implementation mechanism measures mediated provider or child outcomes even though the ICS has been found to mediate outcomes in prior work [41]. Multiple factors could have affected the impact of these mechanisms on outcomes. For example, high variability among providers and across service systems may have led to difficulty finding an effect in our sample. The fact that we tested provider and leader-level implementation strategies in the same sample may have affected the mediation. The interaction between TLI and TIPS may have affected our ability to detect a mediation effect of the ICS. Future research should examine the effects of each TLI component on these mechanisms at varying time points to address these questions.

Although TLI is a brief istrategy found to be feasible in public service systems, the training and coaching were provided by the research team. Future research will be needed to understand how TLI can be scaled up and sustained in public systems with high levels of turnover in both leaders and providers. One potential model could be to train a site-based member of the TLI team to provide ongoing facilitation [57].

## Conclusions

This study provides support for a brief implementation leadership and climate strategy to facilitate successful implementation of autism EBP across two public service systems, children’s mental health and public education. Findings advance the study of implementation mechanisms by examining how and why TLI and other leadership trainings work to facilitate EBP fidelity and identifying a clear need to focus on leaders-level implementation strategies in these systems of care.

## Data Availability

The datasets used and/or analyzed during the current study are available from the corresponding author on reasonable request.

## Abbreviations

AIM HI: An Individualized Mental Health Intervention for Autism
CPRT: Classroom Pivotal Response Teaching
EBI: Evidence-based intervention
ECBI: Eyberg Child Behavior Inventory
EPBAS: Evidence-Based Practice Attitude Scale
ICS: Implementation Climate Scale
ILS: Implementation Leadership Scale
ISS: Implementation Support Strategies
LOCI: Leadership and Organizational Change for Implementation
MI: Motivational Interviewing
PDDBI: Pervasive Developmental Disorder Behavior Inventory
PMI: Provider Motivation Scale
TEAMS: Translating Evidence-Based Interventions for Autism
TIPS: TEAMS Individual Provider Strategy
TLI: TEAMS Leadership Institute
